# An Alzheimer’s disease category progression sub-grouping analysis using manifold learning on ADNI

**DOI:** 10.1038/s41598-023-37569-0

**Published:** 2023-06-28

**Authors:** Dustin van der Haar, Ahmed Moustafa, Samuel L. Warren, Hany Alashwal, Terence van Zyl

**Affiliations:** 1 Academy of Computer Science and Software Engineering, University of Johannesburg, Gauteng, South Africa; 2 Department of Human Anatomy and Physiology, University of Johannesburg, Gauteng, South Africa; 3 School of Psychology, Faculty of Society and Design, Bond University, Gold Coast, QLD Australia; 4 College of Information Technology, United Arab Emirates University, Al-Ain, United Arab Emirates; 5 Institute for Intelligent Systems, University of Johannesburg, Gauteng, South Africa

**Keywords:** Machine learning, Statistical methods, Alzheimer's disease

## Abstract

Many current statistical and machine learning methods have been used to explore Alzheimer’s disease (AD) and its associated patterns that contribute to the disease. However, there has been limited success in understanding the relationship between cognitive tests, biomarker data, and patient AD category progressions. In this work, we perform exploratory data analysis of AD health record data by analyzing various learned lower dimensional manifolds to separate early-stage AD categories further. Specifically, we used Spectral embedding, Multidimensional scaling, Isomap, t-Distributed Stochastic Neighbour Embedding, Uniform Manifold Approximation and Projection, and sparse denoising autoencoder based manifolds on the Alzheimer’s Disease Neuroimaging Initiative (ADNI) dataset. We then determine the clustering potential of the learned embeddings and then determine if category sub-groupings or sub-categories can be found. We then used a Kruskal–sWallis H test to determine the statistical significance of the discovered AD subcategories. Our results show that the existing AD categories do exhibit sub-groupings, especially in mild cognitive impairment transitions in many of the tested manifolds, showing there may be a need for further subcategories to describe AD progression.

## Introduction

Alzheimer’s disease (AD) is the most common form of dementia, and one of the leading causes of death worldwide^1^. AD is currently diagnosed using a clinical evaluation which involves procedures such as physical tests, psychological assessments, and clinical interviews (e.g., collection of demographic and family history data)^2^. While these procedures are adequate for detecting the later stages of AD, they have many limitations. For example, current diagnostic methods are prone to misdiagnosis, are blind to the early stages of the disease, and have varying reliability (e.g., tests vary in performance at different stages of AD)^3–5^. Current clinical diagnoses are not confident and can only be confirmed with an autopsy. It is estimated that significant improvements in diagnostic methods (e.g., detecting the early stages of AD) could lead to advancements in the treatment, understanding, and prevention of AD^6^. Accordingly, there is a critical need to improve current AD diagnostic methods.

Over the last few decades, considerable research has sought to accurately and reliably diagnose AD. These diagnostic studies generally seek to classify the various stages of AD (e.g., significant memory concern and mild cognitive impairment) using multivariate statistical or computational models. For example, it is common for studies to diagnose AD using statistical models that analyse cognitive tests (e.g., mini-mental state examination), brain scans (e.g., magnetic resonance imaging [MRI]), and biomarkers (e.g., beta-amyloid) (see^7^). There has been a shift in recent work towards the detection of the earlier stages of AD, such as mild cognitive impairment (MCI)^8^. Machine learning-based methods have shown some success, but there has been recent success in using deep learning-based methods, such as Alexnet and Resnet18 on MRI data^9,10^. However, there is limited research on deep learning methods applied to other data modalities, such as cognitive tests and biomarkers, and how they can be used to identify AD progression, such as from the control (CN), mild cognitive impairment (MCI) and then AD, along with regressive transitions, such as MCI to CN.

This study proposes using unsupervised approaches such as manifold learning methods such as spectral embedding, multidimensional scaling (MDS), Isomap, t-Distributed Stochastic Neighbour Embedding (t-SNE), and Uniform Manifold Approximation and Projection (UMAP), along with an autoencoder-based embedding of cognitive tests, CSF and other biomarkers to differentiate AD’s various stage transitions better. We show that the t-SNE and autoencoder embeddings can encapsulate more nuanced AD progression stages from cognitive tests and biomarker data and prove the subcategory AD stages that we discovered are valid with statistical analysis (specifically with a Kruskal–Wallis H test). We also show that there is potential for achieving AD progressed stage segmentation without existing priors using clustering in the embedding space, which could be used in a clinical setting for screening patients.

The remainder of this paper is as follows: “Background” outlines early detection AD research, followed by “Literature review”, which describes similar works using statistical, machine learning, or deep learning methods to detect AD. “Experiment setup” explains the study’s methods, key characteristics, and advantages of autoencoders. “Results” outlines the methodology employed for the study, and “Conclusion” presents the results of our study.

## Background

Alzheimer’s disease (AD) is characterised by the impairment of cognitive function in predominately elderly individuals as progressive dementia associated with neurofibrillary tangles, amyloid beta plaques, and neuroinflammation^11,12^. However, this complex neurogenerative disease still has much unanswered questions in the early stages, and it is difficult to differentiate between different stages such as early mild cognitive impairment (EMCI), mild cognitive impairment (MCI), and AD^13^. The difficulty quantifying the structural changes during the transition from the asymptomatic to the symptomatic pre-dementia phase introduces diagnostic uncertainty in the clinical setting. This difficulty, coupled with the co-occurrence of another disease, such as Cerebrovascular disease^14^, makes it difficult for clinicians to automate.

To detect the early stages of AD effectively, an adequate representation is required to encapsulate the changes found in a patient with the potential to progress to AD. Studies have shown some success by analyzing the structural changes in captured data modalities, such as magnetic resonance imaging (MRI), using prior-defined regions of interest or voxel-based morphometry. However, these approaches struggle with spatial reasoning^15^. These methods required well-defined priors, which hindered method development and tiny sample sizes. These limitations drove the exploration of other alternatives, such as machine learning methods, to achieve the feature engineering, segmentation, and classification tasks better and have seen some success in introducing more deep learning-based methods^16–20^.

Other modalities aside from MRI-based data can be used to identify and monitor AD, such as speech^21^, which are less invasive to the patient. Another modality that can be explored when analyzing AD is the demographics, biomarkers^22^, or biosignatures found in the electronic medical records (EMR) for specific AD datasets, such as the Alzheimer’s Disease Neuroimaging (ADNI) database^23^ and OASIS-3^24^. ADNI reports on relatively easily obtainable measurements, such as cognitive scores, genetic risk, vital signs, and plasma biomarkers, beyond the more common MRI-based studies. However, representing these biomarkers or biosignatures in a lower dimensional space while preserving discriminator ability and explainability is challenging.

To better understand how the disease progresses, clinicians can adjust assessment and treatment plans by looking at electronic medical records. They will be able to identify outlier cases more effectively, making this an attractive area of research. Various strategies have been proposed to monitor Alzheimer’s disease (AD) progression over time, many plagued by severe limitations^25^. Much of these limitations are centred around finding better feature presentations or embeddings that can be used to model key phases present during AD progression reliably.

## Literature review

Usually, the collection of data for AD diagnoses has been manually performed by clinicians on-site. However, with the recent adoption of big data techniques in medical practice, tools such as electronic health records could enable new approaches to disease diagnoses. Electronic health records are extensive databases that contain patients’ medical records. Large institutions (e.g., governments) usually run these electronic health records, allowing for the gradual and semi-automatic collection of patients’ medical data^26^. Electronic health records enable clinicians to perform informed and efficient consultations. By expanding the current use of electronic health records, it has been suggested that these medical databases could inform disease diagnoses^27^.

### Alzheimer’s disease detection using classic methods

Using a ventricular enlargement assessment, a clinician can use a standard indicator to differentiate between MCI and AD using a ventricular enlargement assessment^28^. The work shows physiological attributes that allow one to distinguish between key transition points, such as mild cognitive impairment and Alzheimer’s disease, and confirm empirically using a t-test. However, leveraging this approach in an automated manner remains difficult.

After finding attributes that were easier to measure when determining AD progression, a pursuit of models was started, which saw specific covariates, such as age, gender, and the APOE genotype, play a role in AD progression analysis^29^. Although there was significant progress in the research, especially regarding the addition of the ADNI dataset and more multivariate-based approaches, severe limitations were found in methods that model disease progress^25^.

### Alzheimer’s disease detection using machine learning

One way to model this level of differentiation in an automated manner is by using machine learning-based methods. Early works achieve this on the ADNI dataset using sparse logistic regression with stability selection over four years with 15 predictors, which included APOE genotyping, demographic and cognitive measures^30^. The approach performed well and yielded an AUC score of 0.8587 when employing their defined selection approach called biosignature-15, improving their random forest-based works. However, looking at more attributes may be beneficial when exploring more complex models, where attribute count does not contribute to poor performance, as in sparse logistic regression-based methods.

Some studies have suggested that more complex models could diagnose AD using electronic health records. For example, a study by^31^ used machine learning algorithms (such as support vector machines) to predict AD development on electronic health record data. Park et al.^31^ found that their algorithm could predict AD with an accuracy of 71% on a large-scale dataset (the Korean National Health Insurance Service database). While overcoming the burden of data collection related to performing cognitive tests, many of these electronic health record-based models still require better-performing models that can potentially be used in a clinical setting.

Recent work has also shown that it is possible to predict AD transition using classic machine learning methods by first employing a dimensional reduction method (PCA)^32^. They show that it can achieve comparable results to MRI-based method counterparts, and specific attributes contribute more to the overall outcome. However, their principal component analysis (PCA) relies on linear assumptions, potentially diminishing discriminator ability and may hinder model explainability. Green et al.^25^ mentioned that there is value in investigating more latent variable-based approaches to model AD progression. This work shows the first green shoots that prove that it improves performance.

### Alzheimer’s disease detection using deep learning

Many deep learning methods applied to the AD recognition task take a different approach to feature engineering. A representation learning process occurs in the earlier layers of the derived architecture, which differs from the exact feature engineering process seen in previous methods. This representation learning, coupled with pre-trained deep neural networks^33^ has allowed these methods to surpass the performance of current approaches on MRI data^9,10,34,35^, and allowed it to cater to varying modalities^36^. However, as found in the ADNI dataset, a limited amount of research exists that applies deep learning methods, specifically for electronic health records containing cognitive benchmarks, brain measurements, and biomarkers.

Compared to supervised methods, relatively less literature exists for unsupervised AD stage segmentation^37^. The use of methods, such as association, dimensional reduction (such as PCA or ICA^38^, or clustering (including Nonnegative Matrix Factorization-based clustering^39,40^, hierarchical agglomerative clustering^41^, Bayesian clustering^42^ and others^43–45^), applied when analyzing AD stages has potential within a clinical setting. One can identify critical patterns in AD progression to recommend better treatment plans that are more personalized based on patient attributes. The dimensional reduction of electronic health records can map out more comprehensive dependencies and visualize the relationship between different AD progression cases. Lastly, by clustering the data better, we can differentiate between patient categories and determine the level of similarity between a current patient and the derived AD categories. This study addresses this gap by exploring autoencoders as an alternate feature embedding to PCA and analyzing it to determine its discriminator ability for unsupervised AD stage segmentation.

## Experiment setup

In this study, we analyse different manifolds in order to assess their value in analysing AD progression categories using the ADNI dataset. In the experiments we start with the baseline experiment that is related to similar work, then we assess which of the manifold learning methods, namely, Isomap, Spectral, MDS, t-Distributed Stochastic Neighbour Embedding (t-SNE), Uniform Manifold Approximation and Projection (UMAP), and a sparse denoising autoencoder can preserve sufficient information required for reconstruction in order to differentiate AD category progression categories. The learnt embedings are visualised in order to manually inspect emergent AD progression groupings, followed by a clustering assessment using DBSCAN of the best learnt embeddings. Classification accuracy is then assessed using the learnt embeddings and a Support Vector Machine (SVM) trained estimator. Finally a Kruskal–Wallis H Test is done on the orginal attributes for discovered AD progression subgroupings in order validate their significance as new potential AD sub-categories.

### Dataset and data pre-processing

The ADNI dataset is a publicly available secondary dataset from a multisite study that aims to improve clinical trials for the prevention and treatment of AD collected and collated by the private and public sectors across 63 sites in the US and Canada. The data set spans 2004–2021 and contains records of patient visits and the accompanying cognitive tests. The dataset consists of more than 1000 participants with clinical evaluations, neuropsychological tests, genetic markers, AD biomarkers, MRI scans, PET scans and a neuropathological examination if the participant dies and has consented to autopsy. All work was carried out was done following relevant guidelines and regulations set out by the ADNI study and in an ethical manner.

We selected the following fields as fundamental tests.

1. The clinical evaluation data that includes overall health and relevant history
2. All the neuropsychological tests
3. Genetic markers
4. AD biomarkers
5. Neurological, structural measurements

Each individual in the dataset has a baseline visit and several follow-up visits. As a result, the individual patient’s data might be treated as a time series. Considering each patient as a time series is convenient as it provides the mechanism to fill in missing values in the patient’s record. These missing values exist since not every test is performed at every visit. We first remove irrelevant or redundant temporal data, such as duplicated date fields. We also remove variables relating to the protocol. Also, since we are not considering the spatial context, we remove the Site attribute.

All numeric fields are corrected if containing string modifiers such as greater than or less than, and negative values are encoded as missing. Any baseline records without a diagnosis are removed (20 records). Next, we ensure the records are self-consistent, using the baseline values as a ground truth. Then we apply forward filling followed by backward filling for each patient’s time series of visits. Since the diagnosis at baseline and later visits have different resolutions, we encode the visit values in the following way.

1. Both Early Mild Cognitive Impairment (EMCI) and Late Mild Cognitive Impairment (LMCI) are mapped to Mild Cognitive Impairment (MCI).
2. Based on work by^46^, which shows it may not be an appropriate category to represent individuals with cognitive impairment, the subjective memory complaints (SMC) records are mapped to Cognitive Normal (CN).

We one-hot encoded all categorical variables except for the target diagnostic variables, which were removed for fitting or training and used for validation. We fill missing values with the median value for each variable and add a dummy indicator variable to the record where a missing value has been filled. Since the missing values are likely correlated, we use PCA to reduce the dimensionality of the missing value dummy variables. Adding dummy missing value indicators and the subsequent dimensionality reduction resulted in 41 additional dummy variables.

For each record, we add the following target variables:

1. Baseline diagnosis to visit diagnosis (high resolution).
2. Baseline diagnosis to visit diagnosis (low resolution).

### Methods

A popular area of machine learning learning today is that of manifold learning where a succinct or lower dimensional representation is derived that can be useful for downstream tasks. Manifold learning can be divided into linear and non-linear methods^47^. Popular linear methods include principal component analysis (PCA) and multidimensional scaling (MDS) with non-linear methods including Laplacian eigenmaps, Isomap, Local-linear embedding, t-Distributed Stochastic Neighbour Embedding (t-SNE) and Uniform Manifold Approximation and Projection (UMAP). Each of these methods at its core have three attributes: a nearest neighbour search, some way to represent distances or affinities between points and an eigen problem for embedding high-dimensional points into a lower dimensional space^47^.

There are numerous advantages to having lower dimensional representations of data. Not least among these are capturing the most salient features for future analysis and visual analytics. Early attempts focused on embeddings that preserved predefined similarities/relationships in the data, such as adjacency and pairwise distances. Later approaches such as Autoencoders build on the success of deep neural networks and train a neural network to approximate the identity function (trained to reproduce or recontruct its input), the former half of the architecture serve as an encoder and one of the hidden layers (typically before the decoder half) serve as the lower dimensional embedding.

#### t-distributed stochastic neighbour embedding (t-SNE)

To determine the efficacy of the embedding, we employ another embedding, t-distributed stochastic neighbour embedding (t-SNE), to allow us to visualize how well AD stages are segmented. t-SNE is a non-parametric visualization technique that computes pairwise distances affinities according to some perplexity constraint that retains the local structure of the data while revealing some important global structure, such as clusters at multiple scales^48^. It has since become widespread in machine learning as a mechanism for deriving compelling two-dimensional maps of data with many dimensions in a very flexible manner^49^. The study uses it to visualize the derived four-dimensional embedding from the auto-encoder. However, we also use it to visualize the original data to demonstrate the value of the low-dimensional embedding.

#### Uniform manifold approximation and projection (UMAP)

Uniform manifold approximation and projection is a k-neighbour-based graph learning algorithm that competes with t-SNE. It constructs a weighted graph, then transforms its edges to ambient local distance and then derives a lower dimensional representation while preserving topological structure^50^. It deals with higher dimensions and is helpful in contexts that suffer from the curse of dimensionality problems. We chose UMAP as an alternate embedding to confirm findings with t-SNE and to cast light on any global structural differences that can be used for AD stage progression analysis.

#### Sparse denoising autoencoder

An autoencoder is an unsupervised machine learning model that uses the universal approximation capacity of deep neural networks to learn unsupervised lower dimensional representations of data. The embeddings are verified and refined by trying to reconstruct the input from the encoding. The autoencoder learns this dimensionally reduced representation for a data set by training the network to ignore the noise.

Variants of autoencoders exist to compel the learned representations to assume relevant attributes. Variants include regularised autoencoders (Sparse, Denoising and Contrastive) and Variational autoencoders, which can act as density estimators. We are particularly interested in the regularised autoencoders, which are effective in learning representations for subsequent machine learning tasks such as clustering or classification. Autoencoders have been successfully applied to numerous problems, from computer vision to natural language. In the medical diagnostic domain, autoencoders have successfully been employed for medical denoising^51^, medical image searching^52^, feature learning for medical images^53^ and more recently to transform test images to improve predictions^54^.

**Figure 1:**
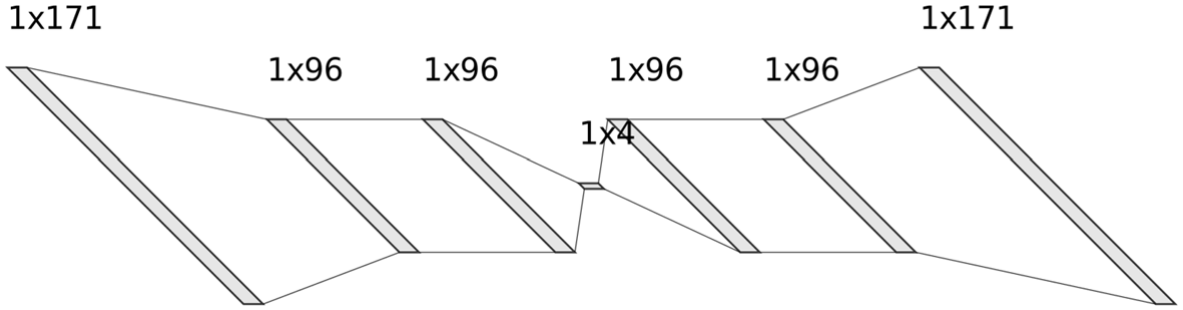
The sparse denoising autoencoder architecture used in the study.

An autoencoder has two main parts: an encoder that maps the input into the embedding and a decoder that maps the embedding to reconstruct the input. The simplest way to replicate an input signal to an autoencoder would be to duplicate the signal. Instead, autoencoders are required to reconstruct the input approximately, preserving only the most relevant aspects of the data by ensuring that the embedding is a squeezed representation of the original input. In this research, we use a Sparse Denoising-Autoencoder. The architecture for the autoencoder can be found in Fig. 1, where the autoencoder consists only of Linear layers with LeakyReLU non-linearities.

Some prior research has combined autoencoders with electronic health records for medical diagnoses. For example, Zhou et al.^55^ applied an autoencoder to two electronic health record data sets. In the first dataset, Zhou et al.^55^ used an autoencoder to predict the length of hospitalisation in individuals with pneumonia. In the second dataset, Zhou et al.^55^ used an autoencoder to classify alcoholics from controls. In turn, Zhou et al.^55^ found that their autoencoder models could predict hospitalisation length with an accuracy of 71.4% and classify alcoholism with an accuracy of 65.1%. In another study, Landi et al.^56^ used a combined autoencoder and convolutional neural network model to distinguish various disorders in electronic health records. Using a clustering algorithm, Landi et al.^56^ could distinguish AD from other disorders and observe the progression of the disease (e.g., classify different stages of severity). However, few studies explicitly focus on AD when using autoencoders and electronic health records for clinical diagnoses.

Moreover, to our knowledge, no study has sought to predict AD progression. Accordingly, this study uses a novel autoencoder model to predict AD development. Specifically, we incorporate electronic health records and an autoencoder-based feature embedding to predict AD progression. We also seek to perform a visual-based analysis that outlines the discriminant ability of our unsupervised autoencoder model. Thus, our aims are as follows: (a) confirm the use of electronic health records for the prediction of AD; (b) use an autoencoder-based feature embedder to predict AD progression, and (c) conduct a visual-based analysis that assesses the discriminant ability of our unsupervised autoencoder model.

Autoencoders are a unique type of deep neural network that learn diagnostic features through the compression and upscaling of data. Through this process of simplification and recreation, autoencoders can learn the key characteristics (features) that underline a phenomenon and, thus, perform diagnoses. Unlike most deep neural networks, autoencoders can be fully automated and are flexible to different data types. Moreover, autoencoders have higher diagnostic accuracy than traditional methods due to their end-to-end feature learning and classification design. The accuracy and efficiency of autoencoders are further aided by their ability to remove noise (unnecessary input) and dimensionally reduce data (compress data). Specifically, dimensional reduction minimises the computational resources required for classification, and noise reduction decreases the chance of errors. These properties make autoencoders specially equipped to process and analyse big data such as electronic health records.

**Table 1:** Hyper-parameter values for autoencoder.

Hyper-parameter	Values
Batch size	512
Learning rate	1e−3
Weight decay	1e−5
Embedding size	7

For the auto encoder, we split the data into training and validation (80–20). We use the 20% validation data to set the hyper-parameters using manual search by minimising the reconstruction loss of the Auto Encoder on the validation set whilst training on the training set. We find the optimal hyper-parameters in Table 1. Finally, once the hyper-parameters are acquired, we use the entire dataset to parameterise an Autoencoder to create the embeddings.

### Manifold embedding evaluation

A fundamental issue with current manifold methods is that there is a lack of methods for embedding quality assessment and many of the evaluation methods are either limited to a specific method (such as Isomap) or are not tolerant of outliers^57^. The study takes evaluates the derived embeddings in a comprehensive way using the following four steps:

1. First an embedding of 2 dimensions is fitted so that it can be visualised for highlighting appropriate local and global groupings present in a qualitative manner.
2. In order to identify groups of similar patients, clustering is performed on the same fitted 2 dimensional embedding to determine if the emergent groupings can be identified in an unsupervised manner.
3. The fitted embeddings (and variants of it) that fared well during clustering are then assessed to determine its potential in a downstream classification task.
4. Lastly, a Kruskal–Wallis H test is performed to confirm subgroupings present in the best embedding to determine the validity of subgroupings based on the original patient data.

#### Embedding visualisation

We are interested in understanding the natural latent space of the embedding within the dataset. We wish to evaluate if the data sits on a lower dimensional manifold embedded in the higher dimensional space. Finding such a manifold would allow us to visualise the data better, identify potential anomalies and outliers, inform future analysis and improve downstream clustering and classification results.

t-SNE was employed for visualising the embeddings since it retains the data’s local and global structure and can be computed relatively efficiently^58^. For the t-SNE parameters, we used parameters commonly accepted in the community and an embedding dimension of two for visualisation. The early exaggeration is set to 12.0 with a learning rate of 200.0, using euclidean distance and Barnes–Hut approximation (with a 0.5 angular size). Varying perplexities were experimented with, and a perplexity score of 130 was found to be the best for the comparison of the selected manifold learning methods.

Uniform manifold approximation and projection (UMAP) was selected as a second visualisation method since t-SNE has been criticised for not appropriately preserving global structure in specific contexts^59^. For the UMAP parameters, we used a 15 local neighbour size, euclidean distance and an embedding dimension of two for visualisation. We also used a learning rate of 1.0 with spectral initialisation, repulsion and spread and a minimum distance of 0.1. The remaining parameters were held at commonly accepted values.

#### Clustering evaluation

In our study, we employed density-based spatial clustering (DBSCAN) because it can group AD categories in the embedding better than partition-based and hierarchical clustering approaches because of its ability to encapsulate arbitrary group shapes and find outliers. Through experimentation, we vary the epsilon or distance threshold that specifies whether points belong to a neighbourhood and the minimum number of data points that constitute a cluster to determine the best parameter combination. We analysed the performance of all the runs and reported on the best-performing learnt manifolds. The metrics we use for this measurement are cohesion in contrast to separation, known as the silhouette score, and the Rand index, which determines the similarity measure between two clusters to their ground truths. The clustering analysis should then be able to determine the potential of an unsupervised approach for key learnt manifolds in the experiment within the ADNI context.

#### Classification evaluation

As an additional test of the learnt embeddings, each embedding is fed into a support vector machine with a radial basis kernel function. Using an 80/20 split to train the model, we benchmark each learned manifold using four common metrics for class predictions: accuracy, precision, recall and f1 score. By evaluating the classification metrics one can determine the potential of the key learnt embeddings within a supervised context.

#### Kruskal–Wallis H test

The Kruskal Wallis test is a non-parametric alternative to the one-way analysis of variance (ANOVA) that tests the ranks of the data instead of the actual data points to determine if there is a significant difference between groups (AD stages in our case)^60^. The null hypothesis assumes that groups are subsets from the same population, and the variance of ranks is computed to derive the H statistic. Since the H closely resembles a chi-square distribution, one can determine if the groups should be in the same population (i.e. if any found subcategories are statistically significant).

The Kruskal–Wallis H test was selected because it is less sensitive to outliers, more compatible with our data and can derive a variance of ranks in a non-parametric manner. Kruskal–Wallis H test can determine if the emergent subcategories are statistically significant and warrant further investigation. However, it is essential to note that the Kruskal–Wallis H test cannot tell us which variables differ between specific groups, only all the groups. The Kruskal–Wallis H test only examines a single variable’s variance across multiple groups. However, more complex learned manifolds may be able to encapsulate more complex relationships that look at more than two variables at once.

## Results

In order to provide a fair assessment of the various learnt manifolds, we include results for the baseline PCA experiment, followed by the various selected learnt manifolds in the study. These include Spectral embedding, Multidimensional scaling, Isomap, t-Distributed Stochastic Neighbour Embedding, Uniform Manifold Approximation and Projection, and a sparse denoising autoencoder.

### PCA baseline results

**Figure 2:**
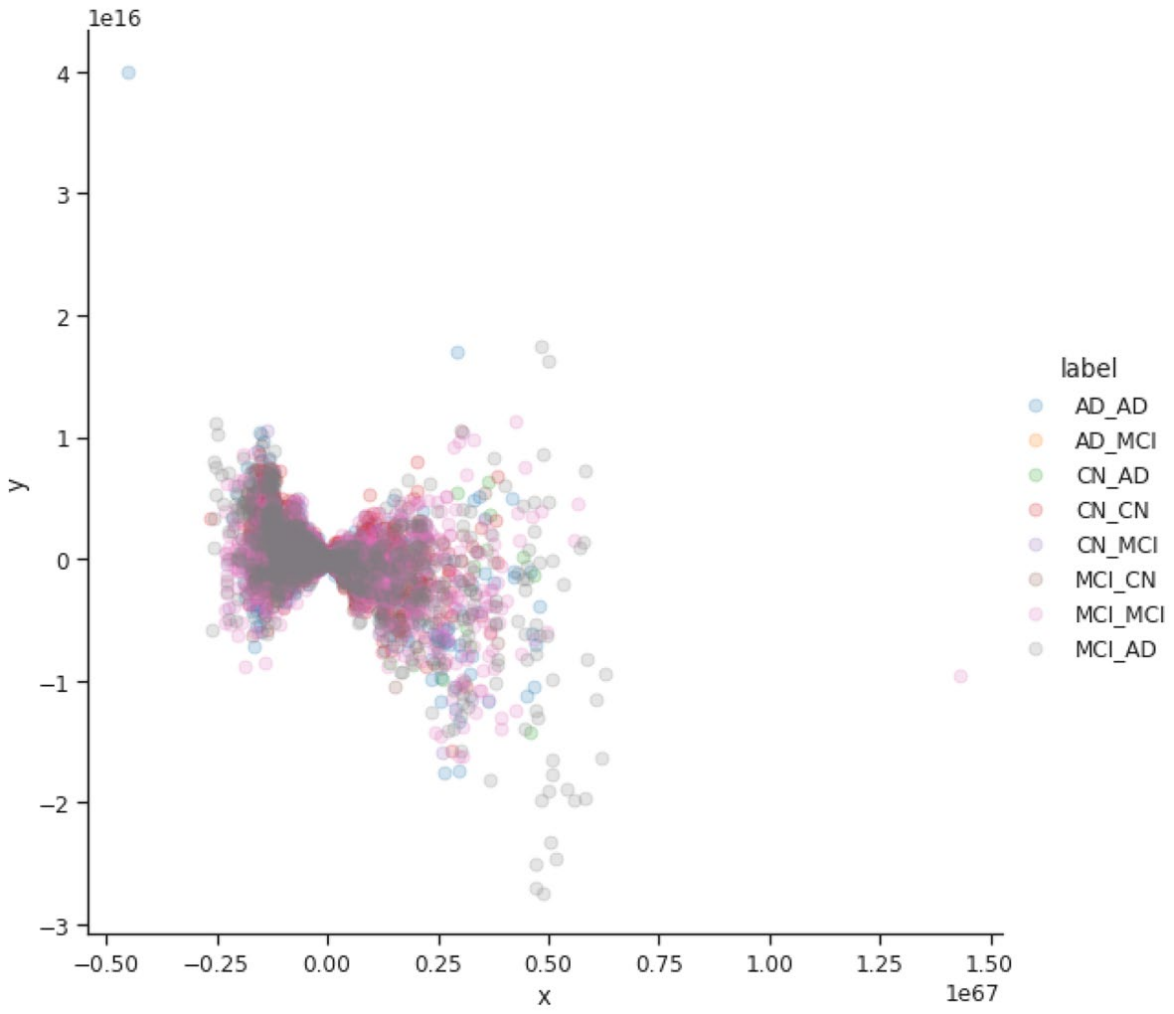
The PCA plot of the manifold built from the pre-processed row subset for the raw, PCA embedding with 2 components.

The first experiment results report on using principal component analysis (PCA) as a lower dimensional manifold inspired by work done by Llera et al.^38^. The PCA results demonstrate the baseline performance from which the other learnt manifold approaches can be compared against. From Fig. 2, we can see that reducing the original data with PCA to two dimensions is not favourable. Upon further exploration, we can see that it has a very low mean explained variance ($3.5\,\times \,10^{39}$), thereby showing that more than 2 dimensions would be more appropriate.

**Figure 3:**
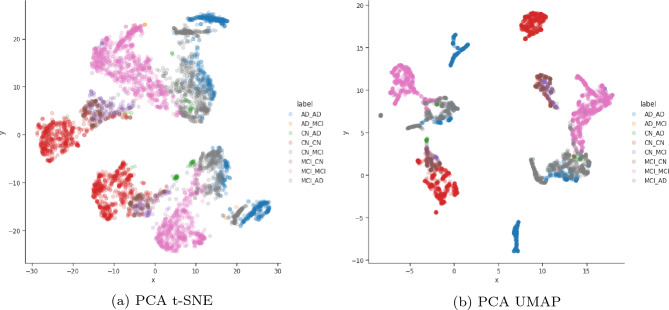
The t-SNE and UMAP plots of the manifold built from the pre-processed row subset for the raw, PCA embedding with 4 components and autoencoder embedding of size 4 and the t-SNE having a perplexity of 130.

The required increase in the amount of components is confirmed in Fig. 3, where one can see that deriving a four dimensional embedding and visualising it using t-SNE and UMAP produce very good results. In both plots each of the AD progression categories are well defined with some categories containing two or more emergent groupings. Following this finding, we explored other manifolds (including t-SNE and UMAP from the raw data) instead of the PCA embedding to see if these emergent groupings are present when using other manifold learning methods.

### Manifold 2-dimensional visualisation results

**Figure 4:**
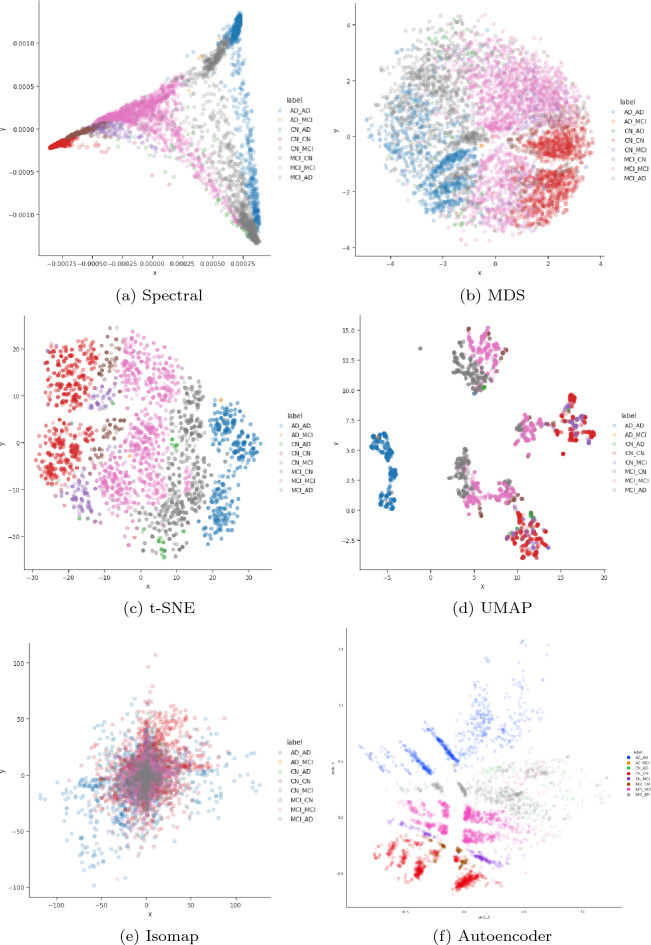
The visualized raw pre-processed Spectral, MDS, t-SNE, UMAP, Isomap, and Autoencoder manifold embeddings with 2 dimensions for the ADNI row subset (i.e. the dataset without the baseline diagnosis).

In the study the manifold learning methods selected were Spectral embedding, Multidimensional scaling, Isomap, t-Distributed Stochastic Neighbour Embedding (t-SNE) on the raw data, Uniform Manifold Approximation and Projection (UMAP) on the raw data, and a sparse denoising autoencoder. For each methods we visualise the different outputs using their two-dimensional embeddings to visualise how local and global structures are formed and ideal candidates are explored further.

For each result that is shown, the optimised version is used, such as using the ideal amount of principal components or the most optimal autoencoder architecture (i.e. using the hyper-parameters shown in Table 1). We also selected the subset with no baseline rows described above that does not contain the baseline diagnosis for a more fair comparison of the works.

From Fig. 4, we can see that some manifold embeddings do not perform as well, such as Isomap. Since Isomap can be viewed as an extension of MDS or Kernel PCA that maintains geodesic distances^61^, it is not an unexpected result. Although, MDS did present groupings of the high level AD categories, the rest of the manifold embeddings had much clearer groupings. The Spectral embedding successfully modelled the AD progression continuum with the exception of the extreme cases that went from cognitive normal to being diagnosed with AD, along with patients reverted to mild cognitive impairment from a AD baseline diagnosis. UMAP provided clear groupings of the high level AD progression categories and similar to the Spectral embedding struggled to encapsulate the abrupt progressions. However, t-SNE and the autoencoder did manage to encapsulate these nuanced progression cases, thereby warranting further investigation.

### Autoencoder results discussion

**Figure 5:**
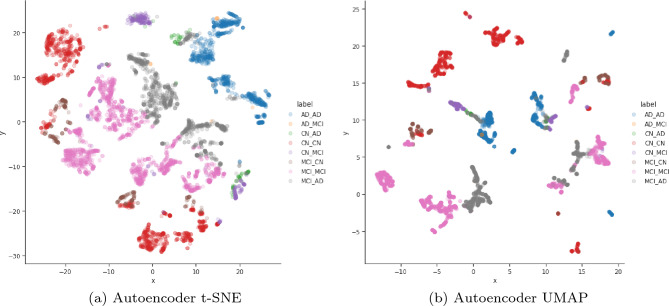
The t-SNE and UMAP plots of the manifold built from the pre-processed row subset for the raw, PCA embedding with 4 components and autoencoder embedding of size 4 and the t-SNE having a perplexity of 130.

Since we t-SNE already optimised the t-SNE parameters to find an ideal embedding (two dimensions with a perplexity of 130, early exaggeration of 12.0, 1000 iterations with a 300 progress cut-off, $\frac{N}{3}$ learning rate and PCA initialisation), we explored increasing the dimensionality of the autoencoder. We found the ideal autoencoder was had an embedding dimension of 4 withe the same parameters outlined in Table 1. When visualising the embedding in two dimensions as seen in Fig. 5, we can see even clearer groupings of the various AD progression categories in both the t-SNE and UMAP plots with the t-SNE showing the problematic progression categories (such as CN to AD) much clearer.

t-SNE preserves the local and global structure. However, there is a tradeoff to retaining local and global structures simultaneously, so we had to carefully monitor the Kullback-Leibler divergence against the outcome during experiments. t-SNE is also susceptible to the perplexity parameter, and after experimentation a constant perplexity value of 130 was selected to visualise the embeddings. The visualisations can be found in Fig. 5, which shows the t-SNE embeddings for the raw row subset data, the PCA and autoencoder embedding. Interestingly, the t-SNE embedding on the pre-processed raw data can still separate the AD categories. However, the PCA and Autoencoder embedding-based visualisation shows they better separate the AD categories. From these visualisations, there is evidence that there may be potential sub-categories of the existing AD categories, which are the focus of the Kruskal–Wallis H test discussed below.

UMAP is considered better at encapsulating global structure differences. However, the density of these clusters is sensitive to minimum distance and local neighbour size, so we had to monitor these parameters and keep them the same across all the embeddings for a fair comparison. As seen in Fig. 5 the UMAP embedding did well at differentiating between the key AD stages and as shown in the separate groupings of samples labelled to be the same AD stage in the PCA and Autoencoder embeddings. This result aligns with the t-SNE embedding visualisations that suggest potential subcategories of existing AD stages, thereby warranting further investigation.

### Clustering results

**Table 2:** The clustering results in a summary of the various learned manifolds using DBScan as a clustering method.

Manifold		Epsilon	Minimum	Silhouette	Rand
Type	Dim’s		Samples	Score	Index
**t-SNE**	**2**	**1.15**	**3**	**0.44**	**0.6**
UMAP	2	0.65	20	0.44	0.26
MDS	2	0.30	30	− 0.05	0.08
Autoencoder	2	0.05	30	− 0.04	0.15

Significant values are in [bold].

**Figure 6:**
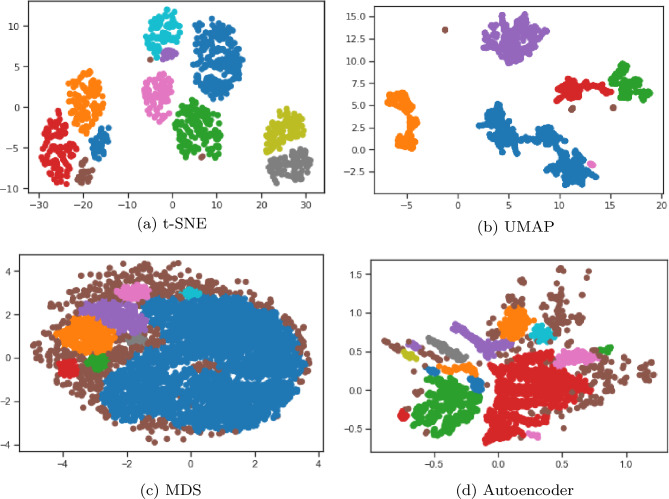
The top four visualized two-dimensional embeddings (t-SNE, UMAP, MDS, and autoencoder) when DB scan clustering is performed.

Table 2 shows the top result for four-manifolds: t-SNE, UMNAP, MDS and the Autoencoder. Interestingly, the t-SNE embedding with a dimension of 2, epsilon of 1.15 and a minimum number of points of 3 relatively good results with a Silhouette score of 0.44 and a Rand index of 0.6. This makes t-SNE a good embedding in our context for downstream tasks such as unsupervised classification or anomaly detection. Figure 6 shows that UMAP, MDS and the Autoencoder could still form good clusters from their learnt two-dimensional embeddings. The figure also confirms the claim that t-SNE exhibits good cluster groupings and shows ten clear clusters that form in the two-dimensional embedding when DBSCAN clustering is performed, two more groupings than the ground truth labels associated with the AD progression categories.

### Classification results

**Table 3:** The classification result summary of the various learned manifolds using a Support Vector Machine with a radial basis function kernel as a classification method.

Manifold		Accuracy (%)	Precision (%)	Recall (%)	F1 score (%)
Type	Dim’s				
**t-SNE**	2	**97.14**	**97**	**97**	**97**
Autoencoder	4	97.14	97	97	97
Autoencoder	2	91.91	90	92	91
UMAP	2	89.35	89	89	89

Significant values are in [bold].

The four best results for the classificatin task are reported in Table 3. The t-SNE with two dimensions achieved an accuracy of 97.14% and precision, recall and F1 score being 97%, thereby further proving t-SNE is an excellent embedding. Interestingly, the second best-performing learnt manifold was the Autoencoder, which had four dimensions, thereby showing value in exploring its performance with embedding dimensions higher than two.

### Kruskal–Wallis H test

**Table 4:** The Kruskal–Wallis H test results of the significant variables, when comparing potential AD subcategories.

Variable	Category1– Category2	Test Stat	Std. Err.	Std. Test	Sig.	Adj. Sig
Age	**EMCI2–EMCI3**	− 194.887	90.365	− 2.157	0.031	1.000
	**EMCI1–EMCI3**	− 189.386	90.292	− 2.097	0.036	1.000
	CN1–EMCI3	− 236.423	86.730	− 2.726	0.006	0.353
	CN2–EMCI3	− 278.049	86.822	− 3.203	0.001	0.075
	LMCI1–EMCI3	269.775	86.730	3.110	0.002	0.103
MMSE	SMC2–CN1	207.421	81.645	2.541	0.011	0.609
	SMC2–LMCI2	219.940	79.253	2.775	0.006	0.303
	SMC1–CN1	184.147	69.702	2.642	0.008	0.453
	SMC1–LMCI2	196.666	66.884	2.940	0.003	0.180
Entorhinal	**AD1–AD2**	− 117.242	54.229	− 2.162	0.031	1.000
	AD1–LMCI2	− 135.935	45.140	− 3.011	0.003	0.143

Significant values are in [bold].

Since the t-SNE embedding visualisations shown in the t-SNE DBSCAN clustering in Fig. 6 show that there may be subcategories present that can further differentiate AD category progressions in patients, a Kruskal–Wallis H test was performed on the t-SNE embedding. The results show that for the t-SNE clusters, 12 of the 44 variables significantly differed across the t-SNE clusters and are the reason they present as subcategories. Table 4 shows that the most significant group differences were confirmed based on Age, DIGITSCOR, TRABSCOR and Fusiform baselines. However, the most significant subcategories were found in the EMCI category when applying Bonferroni significance correction, as shown in Table 4. The computed significance provides evidence that these AD subcategories are valid subgroups differentiated by Age, MMSE and the Entorhinal baseline, thereby warranting further investigation within these groups in a clinician-based study.

## Conclusion

A good representation capable of encapsulating the nuance or non-linearity in differentiating between the early-stage AD categories is a sought-after solution. The shift from statistical to machine and deep learning methods has helped MRI-based studies. However, more work needs to be done in analysing other modalities that are more accessible within a clinical setting. Additionally, more research needs to be done on unsupervised methods that do not require many ground truth labels to work and to analyse AD categories further.

This study shows the value of using autoencoders, t-SNE and UMAP embeddings derived from electronic health records, such as clinical evaluation data, neuropsychological tests and biomarkers for achieving AD progression category segmentation, especially for the early stages. We show that the varying embeddings allow one to analyse the AD progression stages better. The autoencoder embedding is a better representation than the principal component analysis (PCA) used in the literature. Still, the t-SNE embedding performs the best since it encapsulates non-linear relationships and can separate AD categories into more nuanced subcategories. We validate that it is a more appropriate representation by visualising its ability to differentiate between the various categories, using DBSCAN clustering, along with a classification task using a support vector machine and confirm its subcategories with a Kruskal–Wallis H test on the derived cohorts. When Beltran^32^ showed with PCA that there is value in computing a lower dimensional embedding in analysing AD categories. This study shows that the same can be achieved using manifold learning methods, such as t-SNE and UMAP, along with deep learning methods, such as autoencoders, which should be helpful in further downstream unsupervised tasks.

Although our work shows there is value in exploring existing, more recent manifold learning methods for high dimensional AD data with inherent non-linear relationships, future work will demonstrate further there is value in aligning insights found in the embedded space and clinician insights. The alignment should clarify understanding of AD progressions better without compromising too much explainability of method predictions. Another exciting avenue of inquiry is exploring how transformers can be used to derive contextual embeddings within the AD progression context, which can potentially unlock further understanding of categorical-based attributes and generate samples for under-represented AD categories.

## Data Availability

The datasets analysed during the current study are available in the Alzheimer’s Disease Neuroimaging Initiative (ADNI) repository, https://adni.loni.usc.edu/.
